# Novel association of DJ-1 with HER3 potentiates HER3 activation and signaling in cancer

**DOI:** 10.18632/oncotarget.11613

**Published:** 2016-08-25

**Authors:** Shu Zhang, Seema Mukherjee, Xuejun Fan, Ahmad Salameh, Kalpana Mujoo, Zhao Huang, Leike Li, Georgina To'a Salazar, Ningyan Zhang, Zhiqiang An

**Affiliations:** ^1^ Texas Therapeutics Institute, Brown Foundation Institute of Molecular Medicine, The University of Texas Health Science Center at Houston, Houston, Texas, USA; ^2^ Current address: Clinical Research Center, First Affiliated Hospital of Nanjing Medical University, Nanjing, Jiangsu, China; ^3^ Current address: Department of Radiation Oncology, Houston Methodist Research Institute, Houston, Texas, USA; ^4^ Current address: Stemcentrx, Inc., South San Francisco, California, USA

**Keywords:** HER3, DJ-1, anti-HER3 monoclonal antibody, biomarker, cancer

## Abstract

HER3/ErbB3 has emerged as a new therapeutic target for cancer. Currently, more than a dozen anti-HER3 antibodies are in clinical trials for treatment of various cancers. However, limited understanding of the complex HER3 signaling in cancer and lack of established biomarkers have made it challenging to stratify cancer patients who can benefit from HER3 targeted therapies. In this study, we identified DJ-1/PARK7 (Parkinson Protein 7) as a novel interaction partner of HER3 and demonstrated the potential of DJ-1 as a biomarker for anti-HER3 cancer therapy. DJ-1 association with HER3 protects HER3 from ubiquitination and degradation through the proteasomal pathway in breast cancer cells. However, neuregulin 1 (NRG-1) mediated HER3 activation results in a reduced association of DJ-1 with HER3. DJ-1 shRNA knockdown in cancer cells resulted in decreased levels of HER3 and its downstream signaling through the PI3K/AKT and Ras/Raf/ERK pathways. DJ-1 shRNA knockdown cancer cells significantly reduced cell proliferation and migration *in vitro* and tumor growth *in vivo*. Conversely, overexpression of DJ-1 increased HER3 levels and promoted cancer cell proliferation *in vitro* and tumor growth *in vivo*. Notably, cancer cells with high DJ-1 expression showed more sensitivity than DJ-1 knockdown cells to anti-HER3 antibody inhibition. In addition, there was a significant co-expression of HER3 and DJ-1 in tumor tissues of breast cancer patients. Taken together, these results suggest that high DJ-1 expression in breast cancer cells predicts elevated HER3 signaling and may therefore serve as a biomarker for HER3 targeted antibody cancer therapies.

## INTRODUCTION

The EGFR/HER/ErbB family of receptor tyrosine kinases is involved in complex and tightly controlled signaling pathways for the regulation of multiple cellular processes such as cell proliferation and organogenesis. Further studies show aberrant HER signaling results in tumorigenesis [1]. Of the four members in the family, EGFR and HER2 are well-established proto-oncogenes, and a number of cancer therapeutics targeting EGFR and HER2 are currently in clinical use [2]. Due to the lack of intrinsic kinase activity, the importance of HER3 signaling in cancer was not recognized until it was shown to have an important role in acquired resistance to EGFR targeting cancer therapies [3, 4]. Since then, a growing body of work has shown that HER3 plays a key role in sensing perturbations of and maintaining the equilibrium of HER family member dimerization and signaling [5]. Overexpression of HER3 has been reported in multiple cancer types [6, 7] and oncogenic mutations in HER3 were detected in several types of cancer [8, 9]. Therefore, HER3 is emerging as a promising cancer target and multiple anti-HER3 antibodies are currently in clinical trials [10]. However, the lack of biomarkers for HER3-driven cancer poses a big challenge for the clinical development of HER3 targeting antibody therapies [10].

The binding of ligands such as NRG-1 to HER3 induces heterodimerization of HER3 with other HER family receptors, particularly HER2, resulting in HER3 phosphorylation and signaling [1]. Several HER3 interacting proteins such as the E3 ubiquitin ligase NEDD4, Nrdp1, and Nrdp1 regulator USP 8 have been reported for their roles in regulation of HER3 [11–13]. Nevertheless, our understanding of the regulation of HER3 expression and activation remains very limited. DJ-1 is a highly conserved protein and has been implicated in Parkinson's disease [14]. Studies have shown that DJ-1 is involved in cell proliferation and cell cycle progression and has multifunctional properties as regulatory subunit of RNA-binding protein, redox-regulated chaperone, cysteine protease, and transcriptional co-activator [14]. Overexpression of DJ-1 has been reported in many cancer types including breast cancer and various previous studies implicated DJ-1 as an oncogene [15–19]. Despite strong experimental evidence, the molecular mechanisms of DJ-1 in cancer remain obscure and elusive.

In this study, we demonstrated DJ-1 as a novel binding partner of HER3 interacting with the cytoplasmic C-terminal tail of HER3. More importantly, DJ-1 potentiates HER3 signaling strength and predicts sensitivity of cancer cells to the treatment of anti-HER3 antibody. Our work provides a strong rationale for using DJ-1 as a biomarker for measuring response to HER3 targeting cancer therapies.

## RESULTS

### Association of DJ-1and HER3 in cancer cells

**Figure 1:**
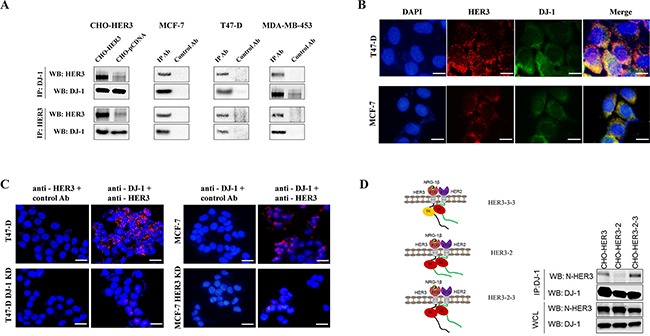
DJ-1 interacts with HER3 **A.** The interaction between HER3 and DJ-1 was examined by co-IP and followed by WB detection. **B.** T47-D and MCF-7 cells were subjected to immunostaining using anti-HER3 (red) and anti-DJ-1 (green) antibodies and fluorescence images were overlapped. Blue indicates the nuclei staining by DAPI and the yellowish color indicates co-localization of HER3 and DJ-1. **C.** The interaction between DJ-1 and HER3 was probed by proximity ligation assay (PLA) in T47-D and MCF-7 cells using anti-DJ-1 and anti-HER3 antibodies. Red fluorescence dots indicate that DJ-1 and HER3 are located in proximity. **D.** CHO cells overexpressing full length human HER3 and two engineered HER2/HER3 chimeras were used for co-IP and WB detection. Antibody against extracellular domains of HER3 is named anti-N-HER3. All experiments were repeated at least three time and representatives are shown.

In an effort to identify HER3 interaction proteins, we conducted a HER3 immunoprecipitation-mass spectrometry (IP-MS) study from lysates of CHO cells that stably overexpress human HER3 (CHO-HER3). The study revealed a panel of HER3 interacting proteins [13]. The IP-MS analysis revealed potential association of DJ-1 with HER3 (Supplementary Figure S1). We first confirmed the interaction between HER3 and DJ-1 in CHO-HER3 cells using Co-IP and WB (Figure 1A). To determine the association of DJ-1and HER3 in cancer cells, we conducted co-IP and WB detection using multiple breast cancer cell lines. Results demonstrated the association between HER3 and DJ-1 (Figure 1A) in all cancer cell lines tested (T47-D, MCF-7, and MDA-MB-453). Immunofluorescence staining also showed co-localization of HER3 and DJ-1 in MCF-7 and T47-D cells (Figure 1B). Proximity ligation assay (PLA), which detects interaction of two molecules within a proximity distance, further confirmed the interaction between DJ-1 and HER3 in MCF-7 and T47-D cells (Figure 1C). However, when DJ-1 or HER3 knockdown (KD) cancer cells (T47-D /DJ-1 KD or MCF-7 /HER3 KD) were tested by PLA, the fluorescent signals were barely detectable (Figure 1C). Taken together, our results using different detection methods consistently demonstrated an association between DJ-1 and HER3 in cancer cells.

To map the intracellular domains of HER3 interacting with DJ-1, we used CHO cells expressing HER3/HER2 chimeric receptors as we described previously [23] and performed IP-WB with antibodies against the extracellular domains of HER3 (N-HER3). CHO cells expressing the HER3/HER2 chimeric receptor that had the HER2 kinase domain and HER3 C-terminus tail (HER3-2-3) did not affect DJ-1/HER3 interaction (Figure 1D). However, replacing the HER3 kinase and the C-terminus tail together with HER2 counterparts (HER3-2) abolished this interaction (Figure 1D). Taken together, these results suggest that the C-terminal tail, but not the kinase domain of HER3 is required for DJ-1/HER3 interaction.

### HER3 activation by NRG-1 negatively impacts the association of HER3 and DJ-1

**Figure 2:**
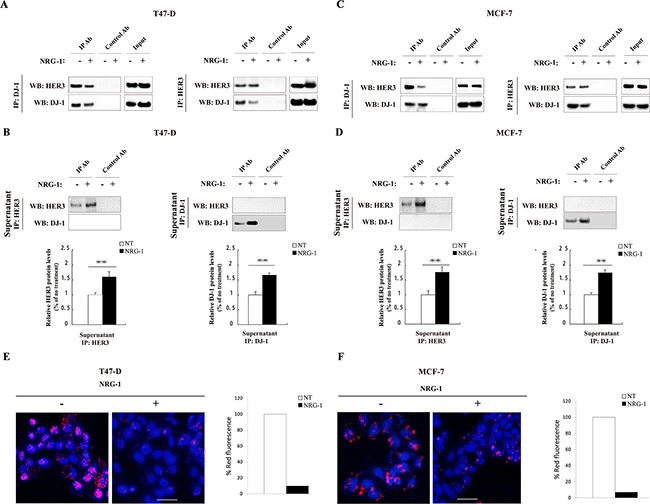
NRG-1 reduces the association of HER3 and DJ-1 **A.** Co-IP was performed with T47-D cells using DJ-1 Ab to pull down the associated HER3 and HER3 Ab to pull down associated DJ-1, and detected by WB. **B.** WB detection of remaining free HER3 or DJ-1 in the supernatants collected from the co-IP study in A. The bar graphs below the WB indicate the signal levels of un-associated HER3 and DJ-1 on the WB. **C.** Co-IP was performed in MCF-7 cells using DJ-1 and HER3 antibodies to detect the association between HER3 and DJ-1. **D.** WB detection of remaining free HER3 or DJ-1 in the supernatants collected from the co-IP study in C using co-IP supernatants of MCF-7 cells. **E.** Effects of NRG-1 on the association of HER3 and DJ-1 by PLA detection. The association of DJ-1 and HER3 (the red fluorescence dots) was quantified. All experiments were repeated at least three time (n=3) and representative images are shown.

NRG-1, as a ligand of HER3, triggers conformational change of HER3 and induces HER3 phosphorylation and signaling [23]. To understand the effect of NRG-1 on the association of HER3 and DJ-1, we investigated the DJ-1/HER3 association in the presence or absence of NRG-1 by co-IP and WB detection using T47-D and MCF-7 cancer cells. The association of DJ-1 and HER3 in the presence of NRG-1 was detected in the first round of co-IP study in T47D (Figure 2A) and MCF-7 (Figure 2C). The disassociation of HER3 and DJ-1 in the presence of NRG-1 was detected in a second round of IP using the supernatants collected from the first round, as shown in Figure 2B, 2D. Results showed that significantly more free HER3 remained in the supernatants of first round co-IP samples where DJ-1 antibody was used to pull down associated HER3 (Figure 2B, 2D). Reciprocally, after immunoprecipitation by HER3 Ab in the first round of co-IP, there was a significantly higher level of DJ-1 remaining in the supernatants from cells with NRG-1stimulation in comparison with the cells without NRG-1 added in the culture (Figure 2B, 2D). To further confirm the effect of NRG-1 on the association of DJ-1 and HER3, PLA was conducted with both T47-D and MCF-7 cancer cells in the presence or absence of NRG-1 stimulation. There was significant reduction of the HER3/DJ-1 association indicated by the fluorescent signal in both T47-D and MCF-7 cells in the presence of NRG-1 in comparison with the untreated cells (Figure 2E, 2F). Taken together, these data suggest that activation of HER3 by NRG-1 triggers the dissociation of HER3 and DJ-1 in cancer cells.

### DJ-1 regulates HER3 mRNA and protein levels

**Figure 3:**
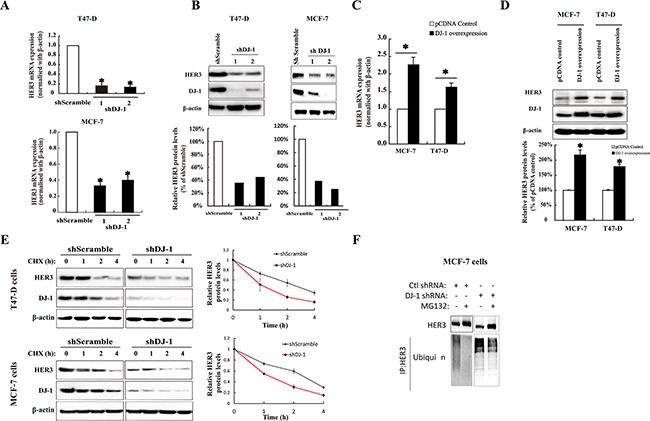
The effect of DJ-1 on HER3 mRNA, protein expression, and protein stability in breast cancer cells **A.** shDJ-1 knockdown in T47-D and MCF-7 cells decreased HER3 mRNA levels. **B.** Stable shDJ-1 knockdown in T47-D and MCF-7 cell lines decreased HER3 protein levels. The bar graphs below the WB image indicate the quantification of WB by ImageJ. **C.** Increased HER3 mRNA levels in T47-D and MCF-7 cell lines with DJ-1 overexpression as determined by qPCR. **D.** DJ-1 overexpression in T47-D and MCF-7 cell lines increased HER3 protein levels by WB and bar graph below the WB image indicates the quantification of WB by imageJ. **E.** DJ-1 shRNA cancer cells had decreased half-life of HER3 protein. Cells were cultured in the presence or absence of cycloheximide (CHX), and cell lysates were collected at indicated times. WB images show the levels of HER3 and DJ-1 and quantitation of the signals by ImageJ is shown in the graphs on the right side. **F.** Increased ubiquitination of HER3 in DJ-1 shRNA cancer cells in the presence or absence of proteasome inhibitor MG132. Cancer cell lysates were subjected to IP using anti-HER3 antibody, and ubiquitinated HER3 was detected with an anti-ubiquitin antibody (P4D1). All experiments were repeated three times, n=3. * indicates p<0.05 and ** indicates P<0.01.

To understand the physiological relevance of the interaction between HER3 and DJ-1, we conducted knockdown of DJ-1 using siRNA transfection of cancer cells. Transient knockdown of DJ-1 by siRNA decreased HER3 at 72 hours post-transfection as compared with the scrambled control (Supplementary Figure S2a). In contrast, we found no changes of the DJ-1 level after knockdown of HER3 in MCF-7 cells (Supplementary Figure S2b). Next, we generated stable knockdown of DJ-1 in MCF-7 and T47D cancer cells. Reduced DJ-1 levels consistently lead to a significant decrease in both protein and mRNA levels of HER3 in the DJ-1 knockdown cancer cells (Figure 3A, 3B). These results indicate that DJ-1 can regulate HER3 at the transcriptional level.

To further investigate the effect of DJ-1 on HER3 expression at mRNA level, we constructed DJ-1 overexpressing cancer cells. Overexpression of DJ-1 in T47-D and MCF-7 cancer cells increased HER3 mRNA levels to approximately two-fold as measured by qPCR (Figure 3C). Similarly, DJ-1 overexpression also resulted in increased HER3 protein levels in both cancer cell lines (Figure 3D).

### DJ-1 modulates HER3 stability

To determine the role of DJ-1 in HER3 protein levels, we treated cells with a protein synthesis inhibitor, cycloheximide (CHX), and monitored the changes of HER3 protein over different time periods. Absence of DJ-1 resulted in a rapid reduction of HER3 protein levels in comparison with the control (Figure 3E). As has been reported by our group and others, HER3 can turn over via ubiquitination mediated proteasomal degradation [13, 24]. Therefore, we examined the effect of DJ-1 knockdown on HER3 ubiquitination mediated degradation. The DJ-1 shRNA cancer cells had increased HER3 ubiquitination in comparison with the scramble control (Ctl-shRNA) in both the presence and absence of an inhibitor (MG132) for ubiquitination (Figure 3F). These results indicate that DJ-1 association with HER3 can increase HER3 stability by slowing down HER3 ubiquitination and proteasomal degradation.

### Reduced HER3 signaling in DJ-1 knockdown cancer cells

**Figure 4:**
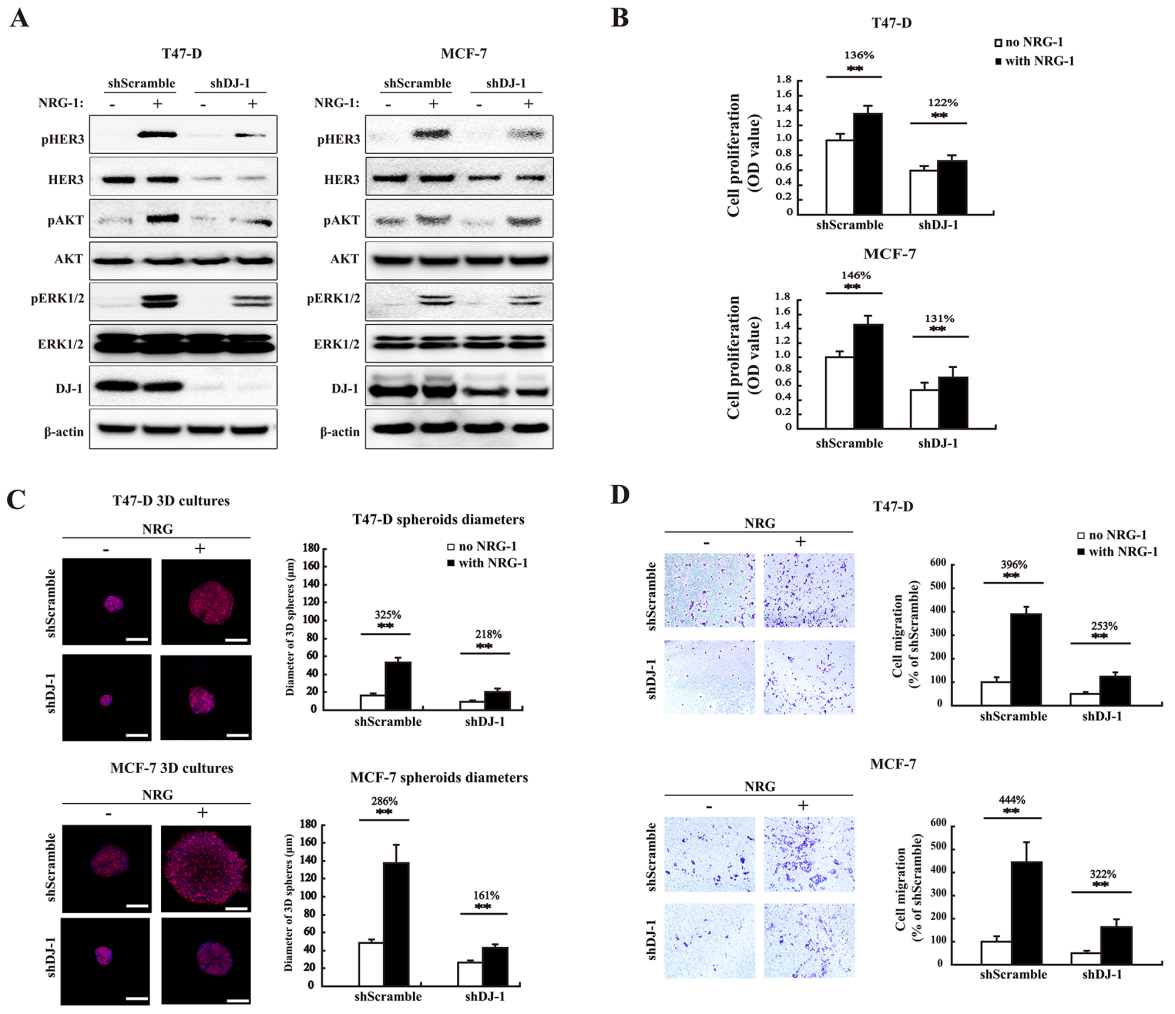
DJ-1 knockdown decreases HER3-mediated signaling, cell proliferation, 3D spheroid growth and migration **A.** WB detection of total HER3, pHER3, AKT, pAKT, ERK1/2, and pERK1/2 in DJ-1 knockdown (shDJ-1) T47-D and MCF-7 cells. **B.** Decreased cell proliferation and stimulation by NRG-1 in shDJ-1 knockdown T47-D or MCF-7 cells. The number on the top of the bars indicates percentage of cell proliferation with NRG-1 by normalizing with the no NRG-1 control as 100%. **C.** Decreased tumor growth in 3D culture in shDJ-1 MCF-7 and shDJ-1 T47-D cells. Scale bars, 100 μm. The sizes of MCF-7 and T47-D 3D spheres were quantified by measuring the diameter of 100 spheres from 10 random fields, n=100. **D.** Reduced cell migration in shDJ-1 T47-D and sh DJ-1 MCF-7 cells. Migrated cells from five random fields were counted. Experiments were repeated at least three times (n=3), ** indicates P<0.01.

It is well established that HER3 can signal through both the PI3K/AKT and the Ras/Raf/MAPK signaling pathways [25, 26]. To determine effects of DJ-1 knockdown on HER3 signaling, HER3 downstream signaling molecules were monitored by WB after cells were treated with NRG-1. As expected, pHER3, pAKT, and pERK1/2 were down-regulated in shDJ-1 cells as compared to the scramble controls (shScramble), while total protein levels of AKT and ERK1/2 were not significantly affected by DJ-1 knockdown (Figure 4A). Similarly, shDJ-1 cells had reduced cell proliferation as compared to the shScramble controls. As expected, DJ-1 knockdown cells also showed a reduced response to NRG-1 stimulation of cell proliferation, 122 % and 131% increase in DJ-1 knockdown cells vs. 136% and 146% in shScramble control for T47D and MCF-7 cancer cells, respectively (Figure 4B). Three-dimensional (3D) cell culture studies also showed that shDJ-1 cells formed smaller cell spheres than those of shScramble cells (Figure 4C). NRG-1 treatment increased cell sphere sizes to 218% and 161% in shDJ-1 T47-D and shDJ-1 MCF-7, respectively; while NRG-1 treatment increased cell sphere sizes to 325% and 286% for shScramble T47-D and MCF-7, respectively (Figure 4C). Again, the data demonstrated that DJ-1 knockdown significantly reduced HER3 activation and promotion of cancer cell proliferation. Similarly, shDJ-1 T47-D and shDJ-1 MCF-7 cancer cells also showed reduced cell migration in the presence and absence of NRG-1 stimulation (Figure 4D). Taken together, these results clearly demonstrate that DJ-1 modulates HER3 levels and impacts HER3 signaling triggered by NRG-1.

### Overexpression of DJ-1 sensitizes cancer cells to anti-HER3 antibody treatment

**Figure 5:**
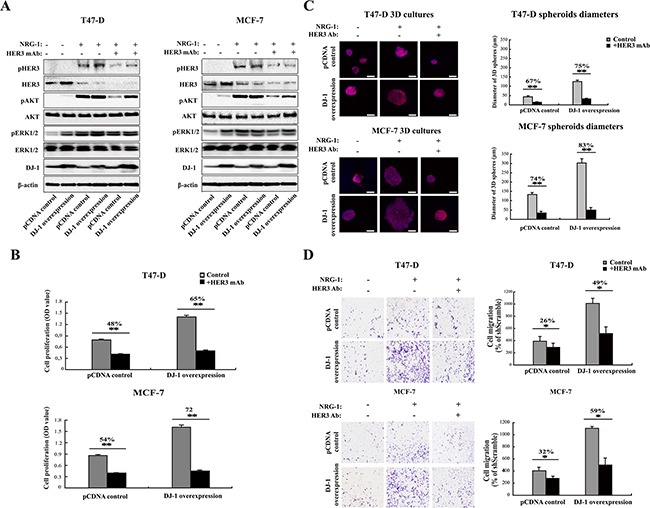
DJ-1 overexpressing cancer cells are more sensitive to anti-HER3 antibody treatment **A.** Inhibition of HER3 signaling by anti-HER3 antibody (HER3mAb) in DJ-1 overexpressing T47-D and MCF-7 cells. **B.** DJ-1 overexpressing T47-D and MCF-7 cells showed increased sensitivity to inhibition of cell proliferation by HER3mAb treatment. The numbers on the top of the bar graph indicate the % of inhibition by HER3mAb treatment, n=4, **P<0.01. **C.** DJ-1 overexpressing T47-D and MCF-7 cells showed increased sensitivity to inhibition of 3D tumor growth by HER3mAb treatment. Scale bars, 50 μm. The sizes of 3D spheres were quantified by measuring the diameters of 100 spheres from 10 random fields. **D.** DJ-1 overexpressing T47-D and MCF-7 cells showed increased sensitivity to inhibition of cell migration by HER3mAb treatment. Migrated cells from five random fields (n=5) were counted and ** indicates p<0.01.

In order to validate DJ-1 as a potential biomarker for HER3 signaling, we determined the effect of DJ-1 expression on HER3 mediated signaling and cancer cell proliferation in response to anti-HER3 antibody treatment. We ectopically expressed DJ-1 in T47-D and MCF-7 cancer cells to elevate DJ-1 expression levels. High DJ-1 expression increased total HER3 as compared to the pcDNA control cells in the absence of NRG-1, and the increase was marginalized in the presence of NRG-1 due to NRG-1 induced HER3 activation (Figure 5A). The result is consistent with the report that DJ-1 overexpression increased HER3 ligand independent signaling [27]. As expected, NRG-1 increased pHER3 levels in DJ-1 overexpressing T47-D and MCF-7 cancer cells, but had no impact on total AKT and ERK1/2 levels (Figure 5A). More importantly, treatment of cancer cells with an anti-HER3 monoclonal antibody (HER3Mab) effectively neutralized the elevated pHER3 and pAKT levels induced by NRG-1 in high DJ-1 expressing and control (pCDNA) cancer cells (Figure 5A). We further examined inhibition of cancer cell proliferation by the HER3Mab in DJ-1 overexpressing cells in comparison with vehicle (pcDNA) control cells. HER3Mab treatment showed 65% and 72% of inhibition of cancer cell growth for DJ-1 overexpressing T47-D and MCF-7 cells, respectively; while inhibition was 48% and 54%, respectively, for pcDNA T47-D and MCF-7 control cells (Figure 5B). In 3D culture studies, DJ-1 overexpressing T47-D and MCF-7 cells formed significantly larger colony spheres than those of the pcDNA control cells in both presence and absence of NRG-1 (Figure 5C). HER3Mab treatment significantly reduced the sphere sizes in both the pcDNA control and DJ-1 overexpressing T47-D and MCF-7 cells (p<0.05), but the effect was more profound in DJ-1 overexpressing cells than in control cancer cells, as indicated by 75% vs 67% reduction in sphere diameters in T47-D cells and 83% vs 74% reduction in sphere diameters of MCF-7 cells (Figure 5C). Anti-HER3Mab also showed significantly more inhibition (49% vs 26% inhibition in T47-D cells, and 59% vs 32% in MCF-7 cells) of cell migration in DJ-1 overexpressing cancer cells as compared to pcDNA control cells (p<0.01, Figure 5D). Taken together, these results indicate that DJ-1 overexpression enhances HER3 signaling and sensitizes cancer cells to anti-HER3 treatment.

### Levels of DJ-1 modulate HER3-driven tumor growth and sensitivity to anti-HER3 antibody treatment *in vivo*

**Figure 6:**
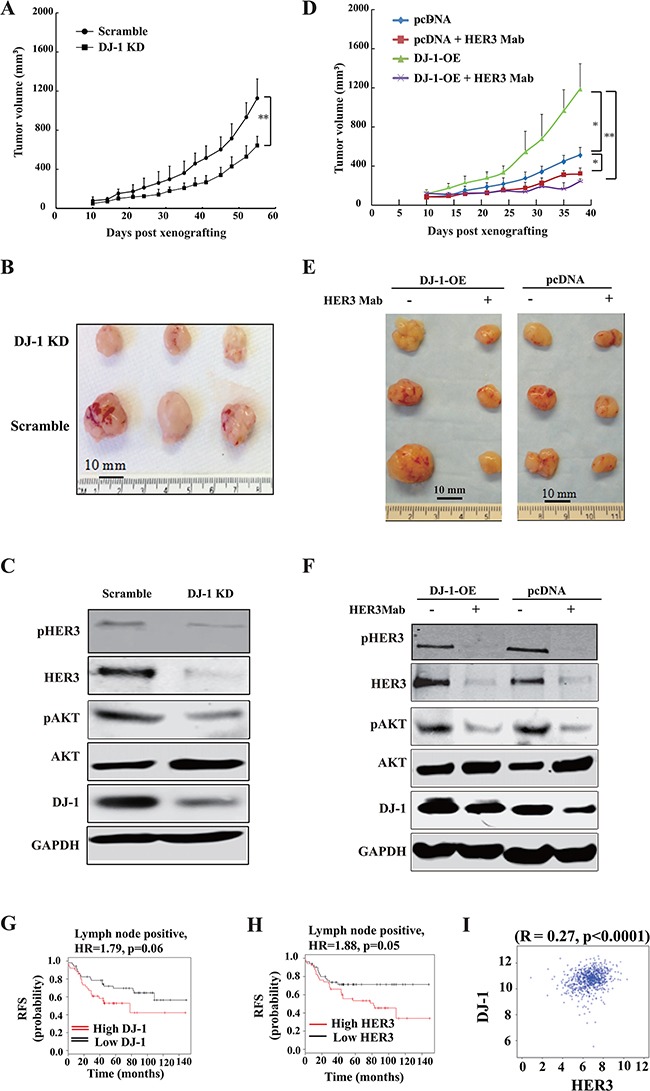
DJ-1 knockdown decreased tumor growth and DJ-1 overexpression increased sensitivity of tumors to anti-HER3 antibody treatment *in vivo* **A-B.** DJ-1 KD MCF-7 cells showed significantly reduced tumor growth, *p*<0.001. **C_D.** DJ-1 overexpression (DJ-1-OE) cancer cells (MCF-7) showed more tumor growth and were more sensitive to HER3mAb inhibition. The graph shows mean tumor volume±standard deviation (s.d.), **p < 0.01, * p < 0.05. **E.** WB detection of DJ-1 and HER3 signaling in DJ-1 KD MCF-7 tumor lysates. **F.** WB detection of DJ-1 and HER3 signaling inhibition by HER3mAb treatment in DJ-1 overexpressing MCF-7 tumor lysates. **G-H.** Correlation of DJ-1 and HER3 expression and relapse free survival (RFS) in lymph node positive breast cancer patients. Kaplan–Meier plots were generated using the data base (www.kmplot.com) forrelapse free survival in patients. HR, hazard rate. I, Significant correlation was found between DJ-1 and HER3 mRNA levels in hormone receptor negative breast cancer patients using Pearson's correlation analysis. Data were obtained from the Cancer Genome Atlas (TCGA) Project and PAM50 defined subtypes.

We examined the effect of shDJ-1 knockdown or overexpression on tumor growth and sensitivity to HER3 antibody treatment in mouse xenograft tumor models. DJ-1 knockdown significantly decreased MCF-7 tumor growth when compared to control tumors (Figure 6A, 6B). In contrast, DJ-1 overexpressing (DJ-1 OE) MCF-7 cells had significantly larger tumors when compared to the pcDNA control (Figure 6D, 6E). HER3Mab completely neutralized the tumor growth as a result of DJ-1 overexpression (Figure 6D, 6E). These results are consistent with the *in vitro* findings that DJ-1 overexpression sensitized cancer cells to HER3Mab treatment. Xenograft tumor tissue *ex vivo* studies confirmed that DJ-1 knockdown decreased levels of total HER3, pHER3, and pAKT in tumors (Figure 6C). DJ-1 overexpressing MCF-7 xenograft tumors had higher HER3 levels without HER3mAb treatment (Figure 6F), but HER3Mab treatment reduced the total HER3 levels, pHER3 levels, and pAKT in tumors from both DJ-1 overexpressing and pcDNA control MCF-7 cancer cells (Figure 6F). The *in vivo* results support the notion that high DJ-1 level promotes HER3-driven cancer progression and sensitizes cancer cells to HER3Mab treatment.

### Association of DJ-1 and HER3 in tumors from breast cancer patients

Analysis of the Cancer Genome Atlas (TCGA) dataset revealed that high expression of HER3 and DJ-1 is associated with poor prognosis in lymph node positive breast cancer patients (Figure 6G, 6H) using the median expression levels as a cutoff for HER3 and DJ-1 expression. We further determined whether the positive correlation between DJ-1 and HER3 levels also exist in clinical cancer patient tumor samples. Interestingly, we found a positive correlation between DJ-1 and HER3 expression in breast cancer (Figure 6I, p<0.0001 using Pearson correlation).

## DISCUSSION

In this study, we report for the first time that DJ-1 interacts with the C-terminal tail of HER3 and modulates HER3 mediated signaling and function in cancer cells. It has been reported that DJ-1 functions primarily as an antioxidant protein with a large array of mechanisms [28]. We showed in this study that DJ-1 modulates HER3 protein stability by delaying the ubiquitination mediated proteasomal degradation of HER3. As DJ-1 has previously been shown to function as a chaperone [14], we speculate based on the data from the current study that DJ-1 functions as a chaperone of HER3.

It has been reported that DJ-1 acts as a positive regulator of AR by impairing the binding of PIASxα (protein inhibitor of activated STAT 2 alpha) [29]. Consistent with previous findings, we also found AR mRNA and protein levels were significantly reduced in the shDJ-1 knockdown MCF-7 cancer cells (Supplementary Figure S3A, S3B). It has been reported that AR mediates HER2 signaling through direct transcriptional regulation of HER3 [30]. Therefore, it is plausible that DJ-1 regulates HER3 transcription via its regulation of AR. However, regulation of HER3 protein levels is not always dependent on DJ-1. Some mechanisms of HER3 protein level regulation are independent of DJ-1 interaction status. Further studies are needed to elucidate the complex molecular mechanism of DJ-1′s role in HER3 homeostasis in cancer cells.

DJ-1 oncogenic function has been demonstrated in a series of published reports and was implicated in cancer cell survival, migration, and invasion by activating the PI3K/Akt pathway and blocking the function of the tumor suppressor gene PTEN [31–34]. In this study, we showed that DJ-1 knockdown results in significant reduction in cancer cell proliferation and migration *in vitro* and tumor growth *in vivo*, while DJ-1 overexpression elevates HER3 levels and promotes NRG-1-dependent HER3 signaling and tumor growth. These oncogenic effects of DJ-1 can be partially attributed to its regulation of HER3 stability and signaling. Our results on DJ-1 interaction with HER3 provide a molecular mechanism for DJ-1 function in cancer promotion. It is well established that ligand binding drives HER3 activation and signaling [35]. NRG-1 has been proposed as a biomarker for clinical development of HER3 antibody cancer therapies [10, 36, 37]. Interestingly, this study revealed that NRG-1 plays a key role in regulating the interaction of HER3 and DJ-1. In the absence of NRG-1 stimulation, DJ-1 associates with HER3 that is in a state of low phosphorylation, and the association between DJ-1 and HER3 protects HER3 from degradation via the ubiquitin-proteasomal pathway. In the presence of NRG-1 stimulation, HER3 exists in a state of high phosphorylation and dissociates from DJ-1. It has been reported that DJ-1 can modulate the AKT-mediated cell survival pathway by negatively regulating the tumor suppressor gene PTEN [32]. Therefore, DJ-1 regulation of HER3 stability and downstream signaling may go through a complex multi-step regulation loop.

There is currently an intense effort to identify biomarkers for clinical development of HER3 targeting antibody therapies [10]. In addition to NRG-1 level in tumors, other proteins that regulate HER3 expression and stability are being investigated as potential biomarkers for HER3 targeting therapies [38]. We found that DJ-1 overexpression sensitizes cancer cells to anti-HER3 treatment. However, the tumor inhibitory effect of HER3mAb treatment is not totally dependent on DJ-1. There are mechanisms independent of the DJ-1 interaction status that influence the effect of HER3mAb treatment. Our recent study identified the E3 ubiquitin ligase NEDD4 as a novel interaction partner of HER3 and a negative correlation between NEDD4 and HER3 levels in prostate cancer, which suggests that the level of NEDD4 may serve as a biomarker in the development of HER3 targeting cancer antibody therapies [13]. This study dissects the role of DJ-1 in regulation of HER3 and elucidates the importance of HER3-DJ-1 interaction in cancer development. High levels of DJ-1 protein have been reported in the sera of breast cancer patients [16, 39]. Preliminary analysis of the TCGA dataset revealed a negative correlation between HER3/DJ-1 expression levels and relapse free survival of the lymph node positive subtype of breast cancer patients. Remarkably, HER3Mab can completely neutralize HER3-driven tumor induced by DJ-1 overexpression. Considering the role of NRG-1 in the HER3/DJ-1 interaction and activation of HER3 signaling, high DJ-1 levels in cancer may predict high HER3 signaling potential when NRG-1 level is high. Therefore, DJ-1 expression may be used as a biomarker in combination with NRG-1 for HER3 activation and response to anti-HER3 therapeutics.

## MATERIALS AND METHODS

### Cell lines, animals and reagents

HEK293T and CHO cell lines were from Life Technologies (Carlsbad, CA). MCF-7, T47D and MDA-MB-453 cells were obtained from ATCC and maintained in a humidified atmosphere of 5% CO_2_ at 37°C, in media suggested by ATCC supplemented with fetal bovine serum (FBS) (HyClone) and penicillin and streptomycin (Life Technologies). Animals (Athymic nu/nu mice) for the *in vivo* studies were from Charles River Laboratories. Monoclonal antibodies against phospho-HER3 (Y1289), AKT, phospho-AKT, ERK, phospho-ERK (42/44), were from Cell Signaling Technology. Antibodies for HER3 and DJ-1 detection by Western blotting were from Abcam. Antibody for detection of total HER3 was from Millipore. NRG-1 was from R&D Systems. Cycloheximide and chloroquine were from Sigma-Aldrich and MG132 from EMD Millipore. The HER3 neutralizing antibody (HER3Mab) was produced in our laboratory and described previously [13].

### Overexpression and stable knockdown of DJ-1 in cancer cells

For stable DJ-1 overexpressing cell line construction, the pcDNA3/FRT vector (GenScript) containing the human DJ-1 was used to transfect cancer cells (MCF-7 and T47-D). Transfected cells were selected by the addition of G418 (20 μg/ml) to culture medium for 3-4 weeks. To generate stable DJ-1 knockdown (KD) cells, Plasmid DNA of shRNA targeting DJ-1 and scramble shRNA in pTRIPz (Thermo Scientific) were amplified in DH5α *E. coli* (Clontech) and lenti-viral particles were produced in HEK-293T cells after 24 h of co-transfection with the shRNA constructs, together with packaging plasmid DNA, psPAX2, and PMD2.G, using lipofectamine (Invitrogen). MCF-7, T47-D, and MDA-MB-453 cells were transfected with the viral particles and cells were selected in RMPI media containing puromycin (4 μg/ml) for 3 weeks as described previously [13].

### Cell lysis, immunoprecipitation (IP), and mass spectrometry

Cell lysis, immunoprecipitation (IP), and mass spectrometry were conducted as reported previously [13].

### Western blotting (WB), reverse transcription and qPCR

Western blotting, reverse transcription and qPCR were carried out as described previously [20]. The following oligonucleotide forward and reverse primers were used for qRT PCR analysis: DJ-1 (5′-GTCATTTGTCCTGATGCCAGC-3′, and 5′-TCAGATAAATTCTGTGCGCCC-3′), HER3 (5′-GGG GAGTCTTGCCAGGAG-3′ and 5′-CATTGG GTG TAG AGA GAC TGG AC-3′), AR (5′-GGAATTCCTGTGCATGAAA-3′ and 5′-CGAAGTTCATCAAAGAATT-3′), GAPDH (5′-CCC ACTCCTCCACCTT TGAC-3′ and 5′-TGTTGCTGTAG CCAAATTC GTT-3′).

### Immunofluorescence (IF)

Cells were fixed with 4% paraformaldehyde for 30 min before immunostaining. Non-specific binding was blocked by incubating cells in a 5% BSA and 0.1% Triton X-100 solution for 1 h at room temperature. Cells were incubated with mouse anti-HER3 monoclonal antibody (1:200) along with rabbit monoclonal anti-DJ-1 antibody in blocking solution overnight at 4°C. After three washes with PBS, cells were incubated with corresponding PE-conjugated goat anti-mouse IgG and FITC-conjugated goat anti-rabbit IgG (1:200) for 2 h at room temperature. Nuclei were stained with DAPI. After three washes in PBS, cells were smeared on glass slides and coverslips were sealed with nail polish. Fluorescent images were acquired using a Carl Zeiss fluorescence microscope (Thornwood).

### In situ proximity ligation assay (PLA)

Cancer cells were grown in 8 well chamber slides to 70-80% confluence. After starvation in FBS free medium for 16 hours, cells were treated with or without NRG-1 for 30 min. Cells incubated with primary antibodies (anti-DJ-1 and anti-HER3) were then incubated with PLA secondary antibodies and substrates (Sigma-Aldrich) as described previously [13]. Fluorescence images were acquired using a Zeiss Axiovert fluorescence microscope (Carl Zeiss Microscopy, Thornwood, NY).

### Cell proliferation and migration assays, and 3D cell cultures

Cell proliferation, migration assay was based on a protocol described previously [13]. For 3D sphere cultures, cells were seeded on top of a Matrigel:medium mixture (1:1) at a cell density of 5000 cells/cm^2^. After 10 days incubation, cells were stained with rhodamine-labeled phalloidin for F-actin and DRAQ5 for nuclei (Molecular Probes) and visualized by confocal microscopy as described earlier [21].

### Cycloheximide and chloroquine treatment

Cancer cells were seeded at a density of 2 ×10^5^ cells in a 6-well plate. After 24 hours, cells were treated with cycloheximide (CHX) at 100 μg/ml, and chloroquine (200μM) for an indicated period of time and lysates were prepared and assayed using WB.

### Mouse xenograft study

Mouse tumor xenograft studies were carried out in accordance with the animal care and use guidelines following a protocol approved by the Animal Welfare Committee (AWC) of the University of Texas Medical School at Houston. DJ-1 shRNA knockdown and scramble shRNA control MCF-7 cells were subcutaneously (s.c.) implanted in nu/nu mice as previously reported [22]. For DJ-1 overexpression and pcDNA control, mice were randomly divided into two groups (n = 3) before antibody treatments on day 10 post implanting cancer cells. HER3Mab or PBS intraperitoneal (ip) injections were performed weekly at a dose of 10 mg/kg for 4 weeks from day 10 to day 38. Tumor growth was measured using a digital caliper and recorded twice a week. Xenograft mouse tumor tissues were collected at the end of *in vivo* study and stored at −80°C for *ex vivo* analysis.

### Statistical analysis

The data are expressed as means ± standard deviation (s.d.) as described in the figure legends. Statistical analysis employed the Student's t-test (two-tail) and *p* < 0.05 is considered statistically significant.

## SUPPLEMENTARY MATERIALS FIGURES


