# Supplementary material for: Novel association of DJ-1 with HER3 potentiates HER3 activation and signaling in cancer
Source: Oncotarget. 2016 Aug 25;7(40):65758–69. doi: 10.18632/oncotarget.11613 (PMC5323190; doi:10.18632/oncotarget.11613)
Supplement: Supplementary file 1 [file oncotarget-07-65758-s001.pdf]

Novel association of DJ-1 with HER3 potentiates HER3 activation and signaling in cancer

SUPPLEMENTARY FIGURES

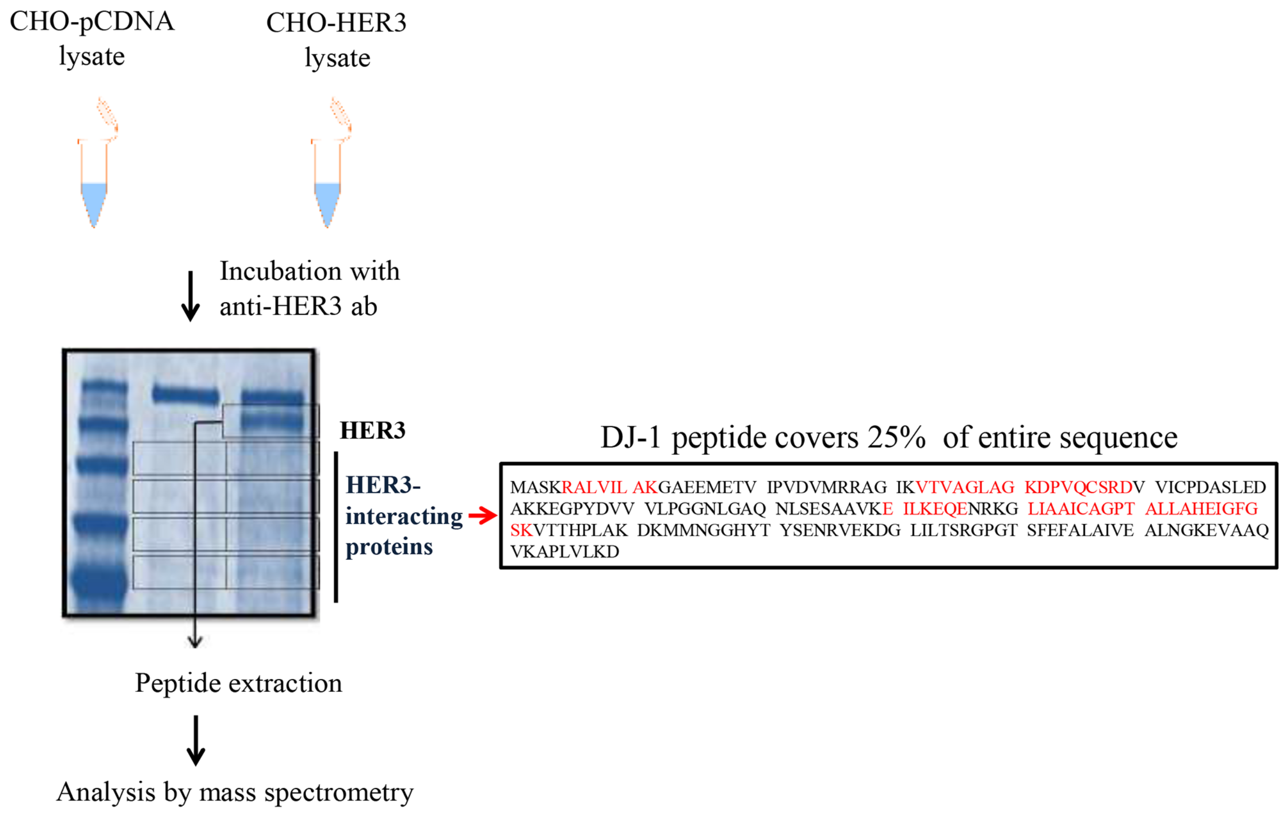

Supplementary Figure S1: Cell lysis, immunoprecipitation (IP), and mass spectrometry. Tryptic digests were individually analyzed on a 6538 UHD Accurate-Mass Q-TOF LC/MS (Agilent Technologies, CA), for shotgun proteomics, and on 6430 Triple-quad LC/MS (Agilent Technologies, CA) for SRM (selective reaction monitoring). Protein hits were obtained using the mascot program with combined data files from each sample run.

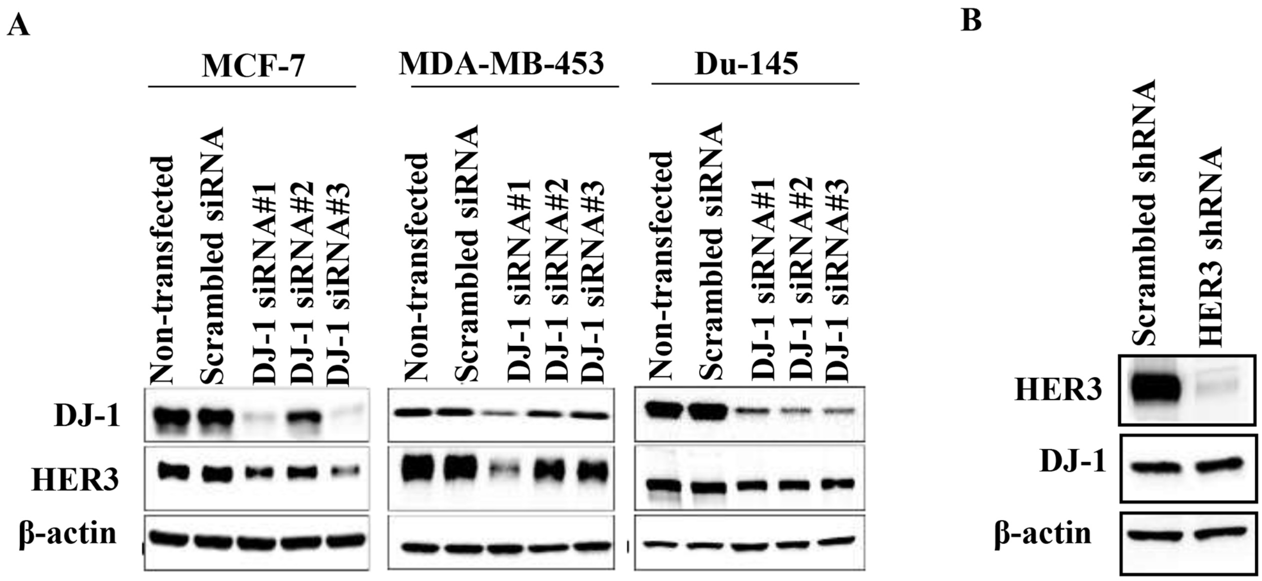

Supplementary Figure S2: A. siDJ-1 knockdown reduced HER3 levels. Three independent siDJ-1 knockdowns in MCF-7, MDA-MB-453 and DU-145 cell lines resulted in significant decrease of HER3 receptor levels as determined by immunoblots. **B.** HER3 shRNA did not reduce DJ-1 protein expression levels in MCF-7 cells.

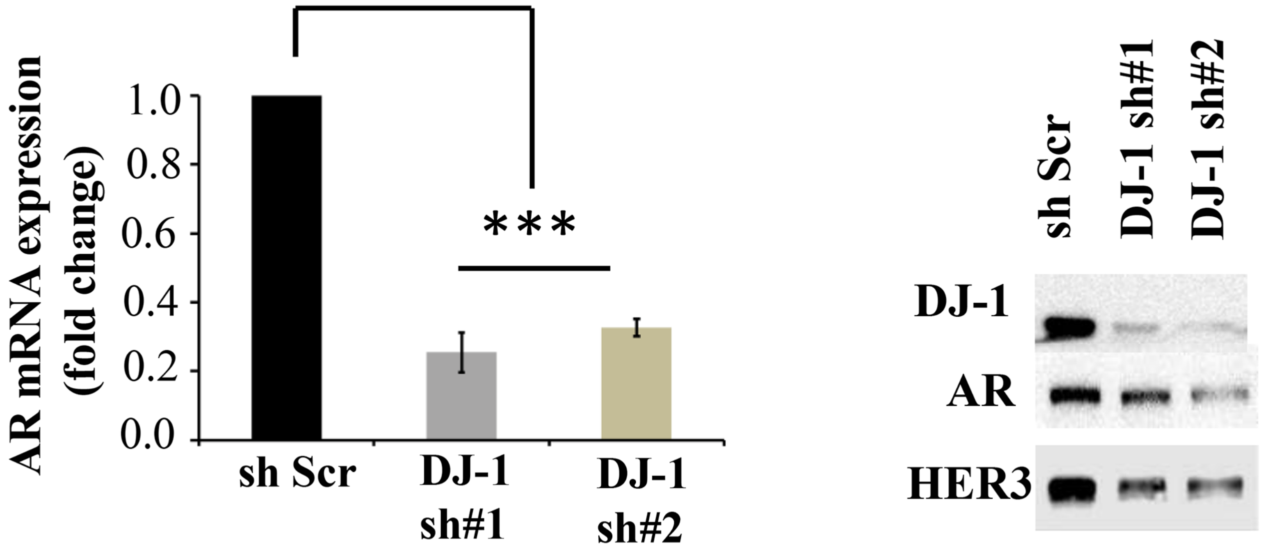

Supplementary Figure S3: Effect of DJ-1 knockdown on AR in MCF-7 cells. A. ARmRNA was analyzed by real-time qPCR, **B.** AR protein expression was detected by Western blotting.
